# Tumor Growth Progression in Ectopic and Orthotopic Xenografts from Inflammatory Breast Cancer Cell Lines

**DOI:** 10.3390/vetsci8090194

**Published:** 2021-09-13

**Authors:** Sara Caceres, Angela Alonso-Diez, Belén Crespo, Laura Peña, Maria J. Illera, Gema Silvan, Paloma J. de Andres, Juan C. Illera

**Affiliations:** 1 Department of Physiology, Veterinary Faculty, Complutense University of Madrid, 28040 Madrid, Spain; sacacere@ucm.es (S.C.); belencre@ucm.es (B.C.); mjillera@ucm.es (M.J.I.); gsilvang@ucm.es (G.S.); 2 Department of Medicine and Surgery, Veterinary Faculty, Complutense University of Madrid, 28040 Madrid, Spain; angalo02@ucm.es (A.A.-D.); laurape@ucm.es (L.P.); pjandres@ucm.es (P.J.d.A.)

**Keywords:** xenograft, ectopic, orthotopic, hIBC, cIMC

## Abstract

Xenografts can grow in immunosuppressed hosts, such as SCID mice, and tumor material can be injected into hosts either ectopically or orthotopically. Choosing the correct model to use is a crucial step in animal research. The aim of this study was to report the differences between ectopic and orthotopic xenografts in tumor progression, metastasis capacity, histological features, and steroid hormone profiles in xenografts from the cIMC (canine inflammatory mammary cancer) cell line IPC-366 and hIBC (human inflammatory breast cancer) cell line SUM149. To achieve this purpose, 40 female mice 6–8 weeks old were inoculated with IPC-366 and SUM149 cells subcutaneously (ectopic models) or into mammary fat pad (orthotopic models). Mice were monitored for tumor progression and appearance of metastases, and generated tumors were analyzed in terms of histological examination and steroid hormone production. The results revealed differences in tumor appearance and percentage of metastasis between ectopic and orthotopic models, which were higher in the ectopic xenografts from both cell lines. However, both models had similar characteristics of tumor progression, histological features, and steroid hormone secretion profiles. We show that the ectopic model can be validated as a good and useful model of tumor development in addition to, not contrary to, the orthotopic model in breast cancer research.

## 1. Introduction

Human and canine inflammatory breast cancer are the most aggressive mammary neoplasms that affects women [1,2,3] and female dogs [4,5]. hIBC accounts for around 6% of human breast cancer diagnoses, presenting poor survival in women, and cIMC is more prevalent than hIBC (approximately 7.6%) [1,4,6]. These diseases are characterized by the invasion of dermal lymphatic vessels by neoplastic cells, which blocks lymph drainage and causes the characteristic edema [1,4,7]. In addition, this type of cancer is highly angiogenic and angioinvasive in both species [1,8,9,10]. Canine inflammatory mammary cancer has been suggested as the best spontaneous animal model for the study of human disease [1,5]. Several human inflammatory breast cancer cell lines have been established in order to study the in vitro mechanisms of this special type of breast cancer such as SUM149 [11,12,13]. Recently, the IPC-366 cell line, the first canine inflammatory mammary cancer triple-negative cell line, has been established and characterized [1,14].

Animal models developed for the study of human breast cancer have been useful tools for refining our understanding of breast cancer progression and metastasis [15,16,17]. Recently, xenografts for cIMC have been established [18]. In general, rodents, such mice, are being used for these studies because they are small in size, breed readily, and can be genetically modified [19]. The advantages of using xenografts are that many of these models are reproducible, are readily available, and a sufficient number can be used in studies to generate valid statistics. The disadvantages are that these models are costlier to run, the stromal component of the tumors is rodent, the hosts are immunodeficient, and most of the time the tumors are grown in a non-natural site [20].

Xenografts can grow in immunosuppressed hosts, such as athymic mice (nu/nu), severe combined immunodeficiency (NOD-SCID, NSG, or HuNSG) mice, or humanized mice, and tumor material can be injected into the host either ectopically (via subcutaneous injection, among others) or orthotopically (inoculation at the site of the primary tumor) [16,17,19]. In breast cancer research, ectopic xenografts are usually performed via subcutaneous and intravenous injections, and orthotopic ones by injecting cancer cells into the mice mammary fat pads [16,17].

Ectopic xenograft models are simple to perform and reproducible and result in a homogeneous tumor histology and growth rate. Accordingly, this type of xenograft is widely used in anticancer drug research [17,19]. In orthotopic xenograft models, the grafted tumor grows in the tissue of origin of the primary tumor. However, complex surgeries are often needed, leading to a limited number of mice used [17,19,20].

In breast cancer research, orthotopic models are the most widely used model. These xenografts better recapitulate the location of the disease and therefore better mimic human cancerous disease [21]. The mammary fat pad is considered the stromal microenvironment of the mammary gland [22], so the inoculation of cancer cells in this component is more similar to human mammary disease [21]. The disadvantages of using this model are that it requires surgery and the number of animals is limited. Another of the models used in breast cancer research is the ectopic model in which cancer cells are injected generally subcutaneously in the mammary chain. The two research models are valid, however, there is little literature on the differences between the two models in breast cancer.

Normal and neoplastic mammary glands are considered endocrine tissues due to the local biosynthesis of steroid hormones [23]. Several studies have shown a strong association between elevated levels of circulating estrogens and their metabolites with an increased risk of developing breast cancer [24]. Furthermore, data from in vitro studies suggest that androgens may also exert antiproliferative and apoptotic effects [25].

Recent studies have shown that the hormonal tumor environment is crucial for tumor development and progression [1]. In addition, male and female mice are capable of reproducing tumors, and their levels of intratumoral hormones will influence tumor progression [1,25]. Therefore, the form of inoculation of tumorigenic material could determine the hormonal microenvironment of the tumor.

This report describes two xenograft models from the hIBC cell line (SUM149) and cIMC cell line (IPC-366). Ectopic and orthotopic xenografts were performed to observe the differences between the two injections in terms of tumor growth, histology, and hormone secretion. Furthermore, this study intended to demonstrate that IMC could be a good animal model for the study of human disease by presenting similarities in tumor growth progression in vivo.

## 2. Materials and Methods

### 2.1. Cell Culture

Canine triple-negative inflammatory mammary carcinoma cell line IPC-366 was obtained from the Department of Physiology of the Veterinary Medicine School of the Complutense University of Madrid (established and characterized in our laboratory [14]). IPC-366 was cultured in Dulbecco’s modified Eagle medium/nutrient mixture F-12 Ham (DMEM/F12) containing 5% fetal bovine serum, 1% L-glutamine, and 1% antibiotic-antimycotic. The human triple-negative inflammatory breast cancer cell line SUM149 was obtained from Asterand, Inc. (Detroit, MI, USA), (RRID: CVCL_3422). SUM149 was cultured in Ham’s F12 (Thermo Fisher Scientific, Madrid, Spain) supplemented with 5% fetal bovine serum, 5 µg/mL insulin, 1 µg/mL hydrocortisone, and antibiotic-antimycotic (Sigma Aldrich, Madrid, Spain). All cell lines were maintained in a humidified atmosphere of 5% carbon dioxide at 37 °C. Cell culture was observed daily via phase-contrast microscopy.

### 2.2. Animals

Forty 6-to-8-week-old female NOD.CB-17-Prkdc scid-RJ mice were used in this study. The animals were housed in flexible-film isolators (Janvier Labs, Madid, Spain) in cages (1–2 animals per cage) in a room with controlled environmental conditions (20–22 °C, 50–55% relative humidity, 10–15 air changes per hour, 12 h/12 h light/dark cycle). Food and water, previously sterilized, were provided ad libitum. The required sample size needed to simultaneously compare the normal means of the groups was determined using the sample size determination module of the Statgraphics Centurion XVI statistical package (Statpoint Technologies Inc., Warrenton, VA, USA). Animals were anesthetized prior to all procedures with isoflurane at 4% for induction and 1.5% to maintain sedation, supplied in a fresh gas flow rate of 0.5 L oxygen/minute, and were observed until fully recovered. Animals were sacrificed using a lethal dose of isoflurane.

Clinical and experimental protocols of this study were approved by the Institutional Animal Care and Use Committee of Complutense University of Madrid, Spain (number: Proex 31/15). All procedures were completed in accordance with the Guide for the Care and Use of Laboratory Animals and conformed to the relevant EU Directive.

### 2.3. Cell Injections

A suspension of 10^6^ IPC-366 and SUM149 cells was implanted subcutaneously (ectopically) into the fourth inguinal mammary gland or orthotopically into the fourth mammary fat pad (both *n* = 20, 10 IPC-366 and 10 SUM149). For orthotopical injections, animals were anaesthetized with isoflurane at 4% and an incision was made medial to the nipple. In order to expose the mammary gland, a cotton swab was used and cell suspension was injected directly into the mammary fat pad with a syringe and a 26-G needle. The wound was closed with a stapled suture.

Mice were inspected twice a week for the development of tumors. When tumors were detected, they were monitored weekly by palpation and measured using calipers. The greatest longitudinal diameter (length) and greatest transverse diameter (width) were measured. Tumor volume was estimated using the formula: volume = (length × width^2^)/2 [26]. When tumors reached a volume of 1.5 cm^3^ (endpoint), blood samples were obtained intracardially and collected in heparin-coated tubes. Prior to this procedure, animals were anaesthetized with isoflurane at 4% for induction and 1.5% to maintain sedation, supplied at a fresh gas flow rate of 0.5 L oxygen/minute. After blood collection, animals were euthanized using a lethal dose of isoflurane. Tumors were harvested at necropsy for subsequent analysis. The appearance of metastasis at the lung and liver were determined macroscopically at necropsy.

The collected tumors were divided into 2 fragments: one fragment for hitological examination was fixed in 4% paraformaldehyde and then embedded in paraffin, and the other fragment was frozen (−20 °C) for hormonal studies.

### 2.4. Histopathology and Immunohistochemistry

Tumors were histopathologically characterized on HE-stained sections following the routine method for histological description of neoplasms [5]. Immunohistochemical characterization of estrogen and progesterone receptors (ER, Ref. M7047, Dako; PR, Ref. 790-2223, Ventana, Oro Valley, AZ, USA) and human epidermal receptor-2 (HER-2, Ref. A0485, Dako, Santa Clara, CA, USA) was performed. Paraffin sections were placed in a PT module, heated for 20 min at 95 °C, and cooled down to 60 °C. Then, slides were rinsed in warm tap water and placed in an automatic immunostainer device (Lab Vision Corp., Fremont, CA, USA) for immunohistochemistry using a peroxidase detection system. After immunostaining, the slides were counterstained with hematoxylin and permanently mounted with Depex. Corresponding negative control slides were prepared by replacing the primary antibody with nonreactive antibody. Slides from human and canine mammary tumors with previously demonstrated reactivity to the primary antibody and tissue internal controls were used as positive controls [5].

For estrogen receptor, progesterone receptor, and HER-2 evaluation, 3+ positive scoring was considered, following the recommended guidelines of the American Society of Cancer Oncology (ASCO).

### 2.5. Steroid Determination in Serum, and Tumor Homogenates

For tumor homogenates, a total of 0.5 g of tumor collected at necropsy was homogenized in phosphate-buffered saline (PBS; pH 7.2) and centrifugated at 1200× *g* for 20 min at 4 °C. Supernatants were collected, aliquoted individually, and frozen at −80 °C until hormones were assayed. Blood samples were centrifugated at 1200× *g* for 20 min 4 °C, and the serum was separated and stored frozen at −20 °C before being assayed.

The hormones evaluated in this study were progesterone (P4), dehydroepiandrosterone (DHEA), androstenedione (A4), testosterone (T), dihydrotestosterone (DHT), estrone sulfate (E1SO4), and 17beta-estradiol (E2). The antibodies used were P4 (C914), A4 (C9111), T (R156), E1SO4 (R522-2), and E2 (C6E91). The antibodies were developed in the Department of Physiology (UCM, Madrid, Spain). DHEA and DHT determinations were performed using a commercially available EIA kit (Demeditech Diagnostic GmbH, Kiel, Germany) following the manufacturer’s instructions.

Determined steroid hormones in tumor homogenates were assayed using previously validated competitive enzyme-linked immunosorbent assay (ELISA), and an amplified ELISA was used for blood samples [26]. Briefly, 96-well flat-bottom medium-binding polystyrene microplates (Greiner Bio-One, Madrid, Spain) were coated with the appropriate purified antibody dilution overnight at 4 °C. Afterward, for competitive ELISA, plates were washed and standards and tumor homogenate samples were diluted in working solution (CWS) and analyzed in duplicate. Plates were incubated at room temperature for 2 h. For amplified ELISA, standards and serum samples were added in duplicate and incubated overnight at 4 °C, then CWS was added to each well and incubated for 4 h at room temperature. For both ELISAs, after conjugate incubation plates were washed, to evaluate the amount of labelled steroid hormones, Enhanced K-Blue TMB substrate (Neogen, Lexington, KY, USA) was added to each well and incubated for an additional 15 min at room temperature. Finally, colorimetric reaction was stopped via the addition of 10% H_2_SO_4_ to each well. Absorbance was read at 450 nm using an automatic plate reader. Hormone concentrations were calculated by means of software developed for this technique (ELISA AID, Eurogenetics, Brussels, Belgium). A standard dose-response curve was constructed by plotting the binding percent (B/B0 × 100) against each steroid hormone standard concentration. All hormone concentrations were expressed in ng/g for tumor homogenates and ng/mL for serum samples.

### 2.6. Statistics

The statistics software used for data analysis was SAS 9.4 (UCM, Madrid, Spain). The results were expressed as means ± SD. For tumor progression analysis (time of palpable tumor, % of tumor engraftment, time of 1.5 cm^3^ volume (edpoint days), and % of animals with metastasis) and hormone determination to compare both cell lines (IPC-366 and SUM149) in each group, the one way ANOVA and Mann‒Whitney rank-sum tests were performed. In all statistical comparisons, *p* < 0.05 was accepted as denoting significant difference.

## 3. Results

### 3.1. Differences in Tumor Appearance Time According to the Manner of Cell Injection

**Figure 1:**
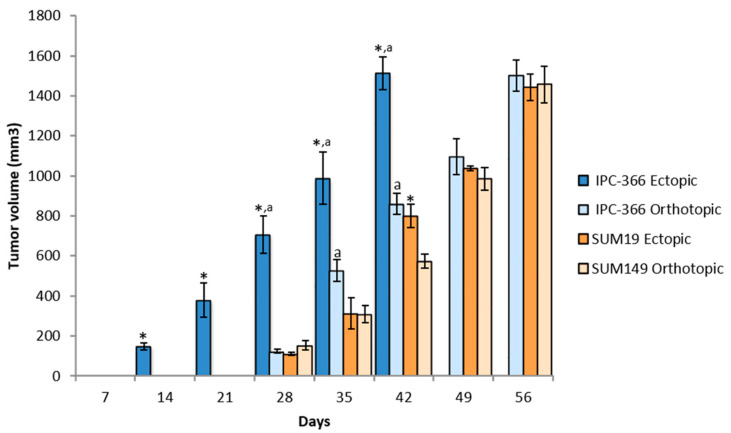
Tumor growth progression in ectopic and orthotopic models. The two injection models of both cell lines showed a fast pattern of tumor growth progression. Bar represents means ± SD * *p* < 0.05; significant differences between ectopic and orthotopic models on each cell line. a; significant differences (*p* < 0.05) between cell lines in each group (ectopic and orthotopic).

IPC-366 and SUM149 cells were injected ectopically and orthotopically in female SCID mice to observe if there were differences in tumor growth (Table 1, Figure 1). When IPC-366 cells were injected subcutaneously, all mice (100%) reproduced a tumor that was palpable approximately 2 weeks after injection (16.64 ± 1.72 days). When cells were injected into MFP, 70% of mice reproduced a tumor approximately 3 weeks after injection (21.40 ± 3.71 days). However, these differences were not statistically significant.

Approximately 4 weeks after injection, with SUM149 cells injected subcutaneously, 80% of mice reproduced a tumor (26.82 ± 2.19 days), and with cells injected into the MFP, 70% of mice reproduced a tumor (30.35 ± 3.47 days), and there was no significant difference in the time palpable tumors were found between the 2 groups.

Regarding differences between cell lines, ectopic injection of IPC-366 resulted in significantly earlier tumor appearance (*p* < 0.05) than SUM149. However, with orthotopic injections no significant differences were found between the two cell lines.

### 3.2. Tumor Progression in Ectopic and Orthotopic Models

After the emergence of tumors, their progression was monitored to observe if there were differences between ectopic and orthotopic models. Tumor progression with IPC-366 and SUM149 cell lines was similar (Figure 1). Both cell lines exhibited rapid growth in vivo, reaching a volume of 1500 mm^3^ approximately 6–8 weeks after injection; it was significantly earlier in the IPC-366 ectopic model (*p* < 0.05), which reached final volume 6 weeks after injection (Table 1).

### 3.3. Occurrence of Metastasis According to the Manner of Cell Injection

These two models developed spontaneous distant metastases (Table 1). No significant differences were found between cell lines in the two models of injection. However, differences in the incidence of metastasis in IPC-366 were found between ectopic (90%) and orthotopic (40%) models, the appearance of metastases in the ectopic model being greater.

### 3.4. Histological Characteristics of Ectopic and Orthotopic Models

**Figure 2:**
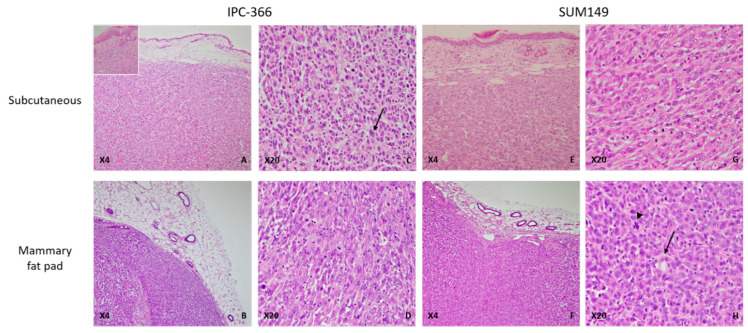
IPC-366 and SUM149 xenotransplanted mice, paraffin sections, H-E. (**A**) IPC-366 ectopic xenotransplanted mice. Neoplastic cells arranged in solid masses separated by a scant fibrovascular stroma infiltrating the adjacent dermis (inset: neoplastic cells infiltrating adjacent dermis). (**B**) IPC-366 orthotopic mice. Unencapsulated and densely cellular mass extending into the adjacent adipose tissue. (**C**,**D**) Ectopic and orthotopic IPC-366 xenotransplanted mice. Tumors are composed of highly pleomorphic cells with marked anisocytosis and anisokaryosis. Binucleated cells are commonly seen (arrow). (**E**,**F**) Ectopic and orthotopic SUM149 xenografted mice. Solid tumors infiltrate the dermis and adipose tissue. No histological differences were found between the types of SUM149 xenografts. (**G**) Orthotopic SUM149 xenograft. Medium to large round cells with a moderate eosinophilic cytoplasm and large nuclei with one or more evident nucleoli. (**H**) Orthotopic SUM149 xenograft. Presence of neoplastic cells with an elongated and empty cytoplasm that displaced the nuclei to the periphery, suggestive of endothelial-like cells (ELCs) (arrow). Atypical mitoses were frequently seen (arrowhead).

The histological examination of tumors from ectopic models revealed highly infiltrating, poorly demarcated, unencapsulated, densely cellular neoplastic growth extending into the adjacent dermis (Figure 1, inset) and striated muscle. Similarly, in the orthotopic models, both IPC-366 and SUM149 xenotransplanted mice had infiltrating, unencapsulated, and densely cellular masses infiltrating the adjacent adipose tissue and compressing the adjacent skin. In both orthotopic and ectopic xenografts, neoplastic cells were arranged in solid masses separated by a scant fibrovascular stroma. The neoplastic cells were medium size, round to oval, with indistinct cell borders and a moderate eosinophilic cytoplasm. The nucleus was medium to large, round to oval, with stippled chromatin, and one to two magenta nucleoli were evident. Anisocytosis and anisokaryosis were marked, the mitotic index was very high, and atypical mitoses were frequently observed (Figure 2).

In addition, some neoplastic cells presented morphological features of endothelial-like cells (ELCs): a rim of elongated, encircled cytoplasm that displaced an elongated nucleus to the periphery was a common finding, suggesting the presence of vasculogenic mimicry (Figure 2).

The presence of emboli in dermal capillaries and marked dermal edema, characterized by colorless spaces that separated dermal collagen fibers, confirmed the histological characteristics of inflammatory mammary carcinoma.

No morphological differences were found between IPC-366 and SUM149 in both orthotopic and ectopic xenografts, so IPC-366 can be considered a good model compared with its human counterpart SUM149 cell line.

Tumors generated by IPC-366 and SUM149 cell lines have common characteristics on the expression of ER, PR, and HER2 and were found to be negative in both ectopic and orthotopic xenografts (Table 2).

### 3.5. Similar Steroid Hormone Secretion in Ectopic and Orthotopic Models

**Figure 3:**
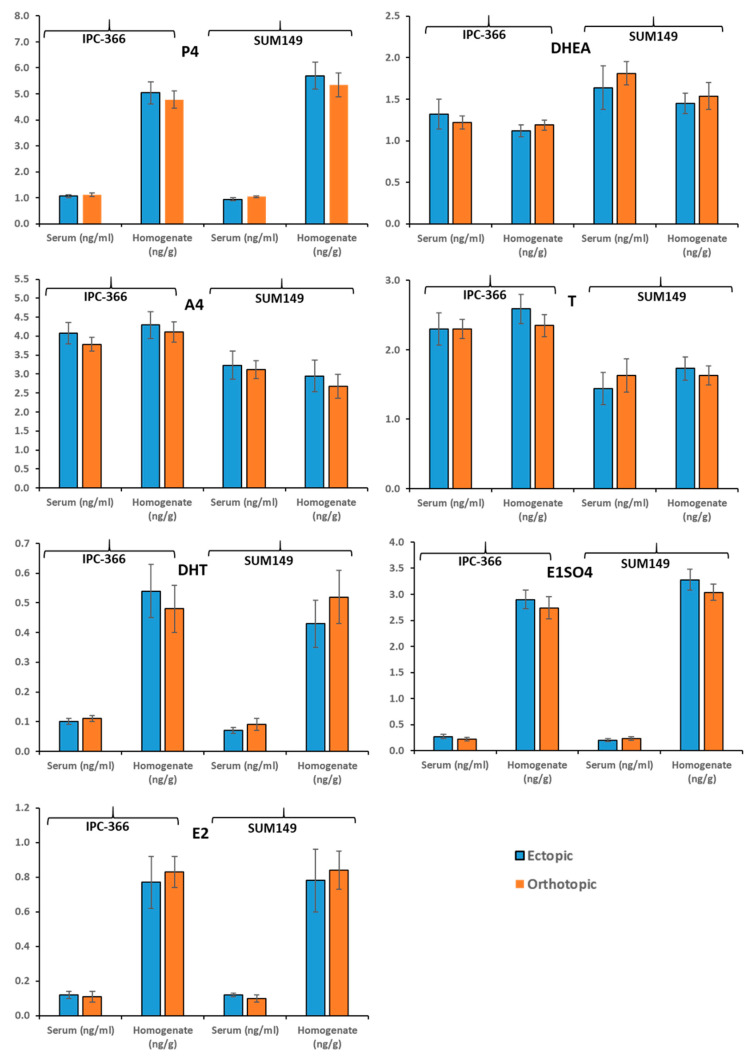
Steroid hormone secretion studied (P4, DHEA, A4, T, DHT, E1SO4, and E2), on ectopic (subcutaneous) and orthotopic (mammary fat pad) models of IPC-366 and SUM149 xenografts.

Figure 3 shows the results of the concentrations of steroid hormones studied (P4, DHEA, A4, T, DHT, E1SO4, and E2), in both serum and tumor homogenate.

No significant differences in the plasma and intratumoral hormonal levels of the human and canine inflammatory carcinoma cell lines (SUM149 and IPC-366) were observed, nor are there differences depending on the type of inoculation (orthotopic or ectopic).

## 4. Discussion

Animal models of human breast cancer are valuable in cancer research for understanding the pathophysiology of cancer, including new target identification [16]. In general, tumor development in murine models is faster and more homogeneous. In the early 1970s, it was demonstrated that human tumor tissues could be successfully grown in athymic nu/nu mice, leading to ectopic tumor xenografts becoming a valuable approach to the study of cancer biology [16,17,19,20]. Nowadays, specific types of tumor models in rodents include ectopic xenografts of tumor-derived cell lines and orthotopic xenografts in which tumor cell lines are implanted into the primary tumor source [16,17,19].

The choice of the type of implantation of tumor cells is a critical step in cancer research, and a comparative study of these models is needed. In tumor models, the murine microenvironment affects the efficiency of engraftment, the rate of tumor growth, and their ability to metastasize [17,19,20,27]. Many breast cancer cell lines have the ability to grow subcutaneously, but ectopic xenograft models have limitations with regard tumor growth location, loss of tumor heterogeneity, and the absence of a specific murine microenvironment, which leads to tumor cells having paracrine interactions with noncancerous cells and tissues [17,18,28,29]. This is why several investigators have moved away from ectopic and use orthotopic xenografts in the MFP. It seems that orthotopic implantation has the advantage that the tumor growth is in the tissue of origin of the primary tumor and facilitating metastatic spread [20,27]. However, this implantation type also has disadvantages, such as the need for complex surgeries, the rodent microenvironment, variable tumor take-up rates, and the long time for primary tumor development [17,18,19,20].

This study intended to determine the differences in tumor characteristics in terms of progression, metastatic capacity, histological features, and hormonal secretion in ectopic and orthotopic models in order to evaluate the capacity of both models for their use in breast cancer research. To achieve this purpose, we decided to use two triple-negative cell lines of canine and human inflammatory carcinoma (IPC-366 and SUM149), which have been shown to have significant tumorigenic potential [30]. cIMC and hIBC are considered to be the most malignant and aggressive subtypes of breast cancer affecting female dogs and humans, respectively [4,6,25].

cIMC has been suggested as a model to study the human disease [4,5,25]. Recently, a triple-negative cIMC cell line (IPC-366) was established as a useful tool for TNBC research [1,14,25]. This study also intended to show that triple-negative cell lines from hIBC (SUM149) and cIMC (IPC-366) resemble each other, sharing in vivo characteristics. The results described above support the statement that cIMC is a good model for studying human disease.

On the other hand, the use of ectopic and orthotopic models in hIBC and cIMC research is limited. There are two murine models (patient derived xenografts) established for human inflammatory breast cancer, MARY-X and WIBC-9 [31,32], and recently a murine model for cIMC was established [18]. Therefore, the use of xenografts from hIBC and cIMC cell lines is crucial for research in this type of cancer.

In this study, the results reveal that with IPC-366 ectopic xenografts, 100% of mice reproduced a tumor 2 weeks post inoculation compared to 70% of mice with orthotopic xenografts, which reproduced a tumor 1 week later. However, SUM149 xenografts did not show significant differences in frequency of tumor appearance (80% ectopic and 70% orthotopic) or time of tumor emergence (4 weeks post inoculation in both). These results are in agreement with the literature [14,25,28]. Thus, ectopic models of cIMC may have an advantage over orthotopic models in that they have higher success rates. Possibly the difference in results between the human and canine models is due to the fact that the IPc-366 cell line presents a tumorogenic and malignancy potential greater than the human SUM149 cell line [30]. Therefore, the ectopic model can be validated as a good and useful model of tumor development in addition to, not contrary to, the orthotopic model. Furthermore, the results showed that IPC-366 and SUM149 grew rapidly in vivo and with tumor progression similar in both ectopic and orthotopic xenografts. Several studies found a correlation between tumor morphology and aggressiveness [4,6]. Agollah and colleagues (2014) showed that the orthotopic SUM149 model grew as multiple nodes/clusters and was capable of producing spontaneous metastasis, which is in accordance with our results in both cell lines [33].

Approximately 40% of hIBC patients have distant metastases to the brain, bones, and lymph nodes [33,34,35]. hIBC and cIMC are capable of spreading into the skin and distant sites through dermal lymphatic vessels [4,33,35]. According to the literature, the metastatic rates of ectopic xenografts are low compared with orthotopic models, in human studies [17]. In this study, the high rates found in both models differed from what is observed in most other tumor types based on literature [17]. A possible explanation could reside in the fact that ectopic subcutaneous injection of breast cancer cells can be performed very near the mouse mammary gland, while ectopic injections of other tumor types, such as brain tumors, are traditionally injected subcutaneously into the hind flank, far from the tissue of origin. In addition, we found a higher percentage of metastasis in the IPC-366 ectopic model than the orthotopic model. However, in SUM149 no differences were found between both models. However, dissemination patterns may vary not only between mice and humans but also among mouse strains [19,20]. Although we found these differences, both models reflect patterns of human and canine disease, such as metastasis, which help in the investigation of inflammatory breast carcinoma and validate the use of both models for a better understanding of breast cancer.

In order to further explore the differences between the ectopic and orthotopic models, we studied the histological characteristics of the tumors generated in both. No morphological differences were found between IPC-366 and SUM149 in orthotopic and ectopic xenografts, so IPC-366 is a good model compared with its human counterpart SUM149 cell line. In both models, neoplastic cells were distributed in solid masses and presented marked anisocytosis and anisokaryosis, characteristics that are similar to the appearance of tumors in the two species studied.

hIBC is predominantly ER-negative, PR-negative, and HER2-positive. It is known that triple-negative breast cancer (TNBC) is highly proliferative and sensitive to chemotherapy and has a poor prognosis [35,36,37]. In our study, the expression of ER, PR, and HER-2 receptors was negative in both the ectopic and orthotopic models, again validating the use of these models in breast cancer research.

It is important that xenograft models preserve inter- and intratumoral heterogeneity [38]. It has been shown that the hormonal tumor environment is crucial for tumor progression and dissemination [1,25]. In order to elucidate whether ectopic and orthotopic models share tumor microenvironment characteristics, an evaluation of their steroid hormone secretion and production profiles was performed. 

In previous studies carried out by our group, possible local synthesis of some steroid hormones was indicated in normal and neoplastic mammary glands in canine mammary carcinoma, and more recently in hIBC and cIMC [1,26]. The formation of sex steroids in peripheral tissues in humans is well documented [39]. The action of progestogens, estrogens, and androgens (produced locally or not) is crucial in neoplastic growth and progression of breast cancer, due to their interactions with specific receptors [1,39]. From the results obtained, it can be seen that the plasma hormone levels (P4, DHEA, A4, T, DHT, E1SO4, and E2) were similar between the ectopic and orthotopic models, and similarly, the intratumoral hormone levels were similar between the 2 models. Thus, the ectopic and orthotopic models presented similar steroid hormone profiles, indicating that both models can be used.

The limitations to this study are that the results are only reproducible for these two human and canine inflammatory carcinoma cell lines and for their use in SCID mice; in other immunosuppressed mouse strains the results may vary. In addition, other factors such as the cell line pass number can influence the engraftment rate.

## 5. Conclusions

Ectopic and orthotopic models with hIBC and cIMC cell lines share characteristics in terms of tumor progression, metastatic rates, histological features, and hormonal secretion profiles, and both are useful for cancer research. Furthermore, the ectopic model can be validated as a good and useful model of tumor development in addition to and not contrary to the orthotopic model.

## Figures and Tables

**Table 1:** Tumor growth parameters of IPC-366 and SUM149 cell lines in ectopic and orthotopic models.

Cell Line	Injection	% of Tumor Engraftment	Time of Palpable Tumor (Days)	Time of 1.5 cm^3^ Volume (Edpoint, Days)	% of Animals with Metastasis
IPC-366 (*n* = 20)	Ectopic (*n* = 10)	100%	16.64 ± 1.72	42.02 ± 2.35	90%
	Orthotopic (*n* = 10)	70%	21.40 ± 3.71	49.81 ± 2.21 *	40% *
SUM149 (*n* = 20)	Ectopic (*n* = 10)	80%	26.82 ± 2.19 ^a^	53.40 ± 4.86 ^a^	80%
	Orthotopic (*n* = 10)	70%	30.35 ± 3.47	51.46 ± 3.67	60%

* *p* < 0.05; significant differences between ectopic and orthotopic models on each cell line. a; significant differences (*p* < 0.05) between cell lines.

**Table 2:** Estrogen receptor (ER), Progesterone receptor (PR), and human epidermal growth factor receptor 2 (HER-2) expression on ectopic and orthotopic xenografts from IPC-366 and SUM149 cell lines.

Receptor	IPC-366 Ectopic	IPC-366 Orthotopic	SUM149 Ectopic	SUM149 Orthotopic
ER	Negative	Negative	Negative	Negative
PR	Negative	Negative	Negative	Negative
HER-2	Negative	Negative	Negative	Negative

## Data Availability

The data that support the findings of this study are available from the corresponding author upon reasonable request.
